# Waning vaccine response to severe COVID-19 outcomes during omicron predominance in Thailand

**DOI:** 10.1371/journal.pone.0284130

**Published:** 2023-05-11

**Authors:** Kannikar Intawong, Suwat Chariyalertsak, Kittipan Chalom, Thanachol Wonghirundecha, Woravut Kowatcharakul, Aksara Thongprachum, Narain Chotirosniramit, Kajohnsak Noppakun, Krit Khwanngern, Worachet Teacharak, Prapon Piamanant, Pannawich Chantaklang, Pimpinan Khammawan

**Affiliations:** 1 Faculty of Public Health, Chiang Mai University, Chiang Mai, Thailand; 2 Chiang Mai Provincial Health Office, Chiang Mai, Thailand; 3 Sansai Hospital, Ministry of Public Health, Chiang Mai, Thailand; 4 Faculty of Medicine, Chiang Mai University, Chiang Mai, Thailand; 5 Nakornping Hospital, Ministry of Public Health, Chiang Mai, Thailand; Carol Davila University of Medicine and Pharmacy, ROMANIA

## Abstract

**Background:**

The COVID-19 pandemic has evolved quickly, with different variants of concern resulting in the need to offer continued protection through booster vaccinations. The duration of enhanced protection with booster doses against severe COVID-19 is still unclear. Understanding this is critical to recommendations on the frequency of future booster doses.

**Methods:**

Utilising a Hospital Information System for COVID-19 established in Chiang Mai, Thailand, we conducted a cohort study by linking patient-level data of laboratory-confirmed COVID-19 cases to the national immunization records, during the omicron predominant period (1 February– 31 July 2022).

**Results:**

Out of 261,103 adults with COVID-19 included in the study, there were 333 (0.13%) severe COVID-19 cases and 190 (0.07%) deaths. Protection against severe COVID-19 was highest with boosters received >14–60 days prior to positive test (93%) and persisted at >60–120 days (91%) but started to wane at >120–180 days (77%) and further at >180 days (68%). The rate of waning differed with age. Those ≥70 years showed faster waning of booster vaccine responses as compared to those aged 18–49 years, who retained good responses up to 180 days. Equivalent risk reduction against severe COVID-19 was seen with all the vaccine types used as boosters in Thailand.

**Conclusions:**

Booster doses provided high levels of protection against severe COVID-19 with omicron, up to 4 months. Repeat boosters will be required to continue protection beyond 4 months, particularly in the elderly. mRNA and viral vector vaccines can be used flexibly to improve booster coverage.

## Introduction

As of 30 January 2023, the Coronavirus disease 2019 (COVID-19) pandemic caused by severe acute respiratory syndrome coronavirus 2 (SARS-CoV-2) has led to more than 670 million confirmed cases and over 6.8 million deaths globally, with almost 5 million cases and 34,000 deaths in Thailand alone [1]. The COVID-19 pandemic has evolved quickly due to the emergence of different SARS-CoV-2 variants of concern. The omicron variant, first described in November 2021, has spread rapidly to become globally dominant [2,3].

The rapid development and deployment of vaccines has significantly reduced the clinical impact of COVID-19 [4,5]. There are seven COVID-19 vaccines currently approved in Thailand [6] and a sustained effort by the government has resulted in 77.6% of the Thai population being fully vaccinated and an additional 38.5% receiving three doses or more as of 2 December 2022 [7]. The primary series vaccinations in Thailand started with inactivated vaccines (Sinovac) [8] in March 2021 followed by ChAdOx1 nCoV-19 (AstraZeneca) [9] in June 2021 and BNT162b2 (Pfizer-BioNTech) [10] in October 2021. Due to challenges in vaccine supply and to manage concerns around the effectiveness and duration of inactivated vaccines, heterologous schedules were implemented from July 2021 onwards including third dose (boosters) mainly with ChAdOx1 nCoV-19 and BNT162b2. Fourth doses (second booster) were implemented more widely beginning in January 2022, using BNT162b2, ChAdOx1 nCoV-19 and mRNA-1273 (Moderna) to address additional concerns around reduced vaccine effectiveness against the omicron variant [11,12].

Although booster doses restore protection against severe COVID-19 outcomes, [13–15] the duration of this enhanced protection in the real world is still unclear. A recent US-CDC study found that among recipients of three doses, the effectiveness against COVID-19–associated hospitalizations declined from 89% among those vaccinated within the past 2 months to 66% among those vaccinated 4–5 months earlier [16]. Similar results were seen in Brazil, where protection waned after 3–4 months from the last booster vaccine, and the decline was more rapid with homologous schedules as compared to heterologous schedules [17]. Previous studies examined waning of booster vaccine effectiveness against severe outcomes in populations with predominantly homologous schedules [15,16,18] and only a small number of studies have examined heterologous schedules [17,19,20].

Most countries in Asia, including Thailand, implemented heterologous vaccination schedules early in the pandemic often incorporating inactivated vaccines in the primary series. It is therefore critical to understand the duration of enhanced protection offered by heterologous booster vaccines to better inform recommendations relating to the frequency of future booster doses.

## Materials and methods

### Study population

The current study draws on a unique hospital information system (HIS) established in Chiang Mai, located in Northern Thailand, with a population of 1.6 million. Adults with laboratory confirmed COVID-19 between 1 February– 31 July 2022 were included in the study. To understand the epidemiology of circulating SARS-CoV-2 variants, the ministry of health performs molecular testing on a random sample of COVID-19 positive cases every month. Test results from northern Thailand revealed that BA.1 omicron sub-lineage accounted for >90% of the cases in February. BA.2 was first detected in March and BA.4/BA.5 was detected from mid-June onwards. Subsequently, the BA.4/BA.5 sub-lineage spread rapidly and accounted for >90% of cases by mid-August [2].

Non-Thai residents and migrants were excluded from the study as the vaccination data and outcome capture for this group was incomplete. Cases with missing age were also excluded. The patient selection flow is presented under **S1 Fig**.

### Data sources

We have previously published the details relating to creating and implementing the information systems used in this study [21]. In brief, all COVID-19 cases detected in Chiang Mai province are reported into the web based HIS of Chiang Mai Provincial Health Office (CMC-19 HIS). During the study period, reporting of all COVID-19 cases was mandatory under the Communicable Disease Control Act. When a COVID-19 case is detected, either at screening centers, hospitals or out-patient clinics, the healthcare staff enter the patient details, including laboratory results into the CMC-19 HIS under a unique ID. Data on severity and progression of the disease including requirement of ventilatory support and treatments are recorded in each hospital’s information system, which is linked with CMC-19 HIS. Deaths which occur within the province are reported to Chiang Mai Provincial Health Office and are routinely updated in CMC-19 HIS.

All national vaccination records are centrally captured in the Ministry of Public Health Immunization Centre (MOPH IC) database maintained by the Ministry of Public Health, Thailand.

### Ethical approval statement

The study was conducted on routine data collected as part of the national COVID-19 response under the Communicable Disease ACT (B.E. 2558) and was exempted from ethics review and informed consent.

### Study design

We conducted a retrospective cohort study on Thai residents aged 18 years or older, with a laboratory confirmed SARS-CoV-2 infection between 1 February and 31 July 2022. Date of first positive SARS-CoV-2 test served as the index date. Reinfections, defined as a positive SARS-CoV-2 test at least 90 days prior, accounted for <0.5% of this cohort.

Baseline clinical characteristics and SARS-CoV-2 test details were extracted from the CMC-19 HIS. The types of COVID-19 vaccines, and dates of vaccination were extracted from MOPH-IC immunization database.

“Severe COVID-19” was defined as requiring invasive mechanical ventilation (IMV) during hospital admission, or death within 30 days of a positive SARS-CoV-2 test. Records of all subjects were followed till death, or up to 30 days from first positive test, whichever was earlier. The severe outcome capture for the study population is near complete as the clinical information of all hospitalised COVID-19 cases of the 26 public and 8 private hospitals in Chiang Mai province, including the only two tertiary care referral hospitals providing IMV support in Chiang Mai, are entered into a single electronic data capture platform.

### Statistical analysis

Descriptive statistics are reported separately for the subjects with and without severe COVID-19 outcomes to understand the differences in baseline characteristics between the groups. Continuous variables are summarized as mean and standard deviation (SD) for normally distributed data or median and interquartile range (IQR) for skewed data. Categorical variables are summarized as frequency and percentages. Between group comparisons for continuous variables were done using *t-test* (normally distributed data) or Mann–Whitney *U* test (skewed data). Between group comparisons for categorical variables were done using chi-squared test or fisher’s exact test, as appropriate.

Cox proportional hazards regression was used to estimate the hazard ratios (HRs) for severe COVID-19 and mortality outcomes. For the analysis with severe COVID-19 as outcome, the follow up period was taken from the first positive SARS-CoV-2 test date and censored at the earliest of: date of first starting IMV, date of death or 30 days from first positive test date. For the analysis with death as outcome, the follow up period was taken from the first positive SARS-CoV-2 test date and censored at the earliest of: date of death or 30 days from first positive test date. If the outcome occurred on the first positive SARS-CoV-2 test date, the follow-up time was taken to be 0.5 days.

Age, gender, calendar day of test (in weekly units), vaccination status, and time since last vaccination were added as factors in the regression model to estimate adjusted HRs (aHR) and the 95% confidence interval (95% CI) for severe COVID-19 and mortality outcomes separately. The vaccination status was categorised as partially vaccinated (received only one dose), primary series (received two recommended doses), three-dose (received primary series and one booster dose), and four-dose or more (received primary series and two or more booster doses) with the unvaccinated group serving as reference group. To examine waning of vaccine response, subjects who were vaccinated were sub-grouped using time specific indicators defined by two-month intervals of time since vaccination (≤14 days, 14 to 60 days, >60 to 120 days, >120 to 180 days and >180 days) for each vaccine status. Due to the limited number of events, subjects who received four-dose or more were combined with the three-dose group during analysis (three-dose or more). A separate sensitivity analysis was done among unvaccinated, partially vaccinated and three-dose group after excluding subjects who received four-doses or more to assess if the estimates were different. Separate analyses were done to examine if the waning vaccine response differed by age groups (18–49 years, 50–69 years, and ≥70 years) and by booster vaccine types (Pfizer-BioNTech, Moderna and ChAdOx1 nCoV-19).

All statistical analyses were be conducted using stata (version 15.0 SE, College station, TX: StataCorp LP). Significance tests are 2 sided and a *p*-value <0.05 was considered statistically significant.

## Results

### Baseline clinical characteristics

There were 359,956 COVID-19 cases reported between 1 February– 31 July 2022, in Chiang Mai province, Thailand. After applying the exclusion criteria, 261,103 (73%) Thai residents above 18 years of age with complete data were included in the final analysis **(S1 Fig).** Severe COVID-19 outcomes and deaths were observed in 333 (0.13%) and 190 (0.07%) subjects respectively.

As compared to subjects without severe COVID-19 outcomes, those with outcomes were nearly 30 years older and were more likely to be male **(Table 1)**.

**Table 1:** Comparison of clinical characteristics of adult COVID-19 patients with and without severe outcomes during omicron predominance (1 Feb 2022–31 Jul 2022) in Chiang Mai, Thailand.

Variable	Without severe COVID-19 outcome	With severe COVID-19 outcome	*p-value*
**Number (%)**	260,770 (99.8)	333 (0.13)	-
**Age, years**	** **	** **	*<0*.*01*
Median (IQR)	39 (27–55)	71 (59–82)	
**Age group, n (%)**	** **	** **	*<0*.*01*
18–29	78817 (30.2)	11 (3.3)	
30–39	56054 (21.5)	14 (4.2)	
40–49	42669 (16.4)	20 (6.0)	
50–59	36037 (13.8)	44 (13.2)	
60–69	31368 (12.0)	63 (18.9)	
≥70	15825 (6.1)	181 (54.3)	
**Gender, n (%)**	** **	** **	*<0*.*01*
Male	115053 (44.1)	188 (56.5)	
Female	145717 (55.9)	145 (43.5)	
**Vaccination Status, n (%)**	** **		*<0*.*01*
Unvaccinated	25250 (9.7)	172 (51.7)	
Partially vaccinated	3692 (1.4)	10 (3.0)	
Primary vaccine series	118675 (45.5)	107 (32.1)	
Vaccinated three doses	97428 (37.4)	40 (12.0)	
Vaccinated four doses or more	15725 (6.0)	4 (1.2)	
**Type of primary vaccine series, n (%)**	**n = 118,675**	**n = 107**	*0*.*03*
Sinovac/Sinopharm-ChAdOx1 nCoV-19	47013 (39.6)	28 (26.2)	
Sinovac-Sinovac or Sinopharm-Sinopharm	16523 (13.9)	14 (13.1)	
ChAdOx1 nCoV-19-ChAdOx1 nCoV-19	3595 (3.0)	7 (6.5)	
Pfizer-BioNTech-Pfizer-BioNTech	13862 (11.7)	13 (12.1)	
ChAdOx1 nCoV-19-Pfizer-BioNTech/Moderna	32110 (27.0)	36 (33.6)	
Sinovac/Sinopharm-Pfizer-BioNTech/Moderna	593 (0.5)	1 (0.9)	
Moderna-Moderna	4978 (4.2)	8 (7.5)	
**Time since last dose of primary series, n (%)**	**n = 118,675**	**n = 107**	*<0*.*01*
≤14 D	436 (0.4)	3 (2.8)	
>14 to 60 D	5761 (4.9)	5 (4.7)	
>60 to 120 D	47379 (39.9)	34 (31.8)	
>120 to 180 D	42308 (35.6)	30 (28.0)	
>180 D	22791 (19.2)	35 (32.7)	
**Type of third vaccine dose, n (%)**	**n = 97428**	**n = 40**	*0*.*75*
Pfizer-BioNTech	48465 (49.7)	21 (52.5)	
ChAdOx1 nCoV-19	27614 (28.3)	13 (32.5)	
Moderna	21265 (21.8)	6 (15.0)	
Other	84 (0.1)	0 (0)	
**Time since third vaccine dose, n (%)**	**n = 97428**	**n = 40**	*0*.*09*
≤14 D	5099 (5.2)	3 (7.5)	
>14 to 60 D	27862 (28.6)	7 (17.5)	
>60 to 120 D	41362 (42.4)	14 (35.0)	
>120 to 180 D	16202 (16.6)	10 (25.0)	
>180 D	6903 (7.1)	6 (15.0)	
**Type of fourth vaccine dose** * **, n (%)**	**n = 15330** *	**n = 4**	*-*
Pfizer-BioNTech	6836 (44.6)	3 (75.0)	
ChAdOx1 nCoV-19	803 (5.2)	0 (0)	
Moderna	7679 (50.1)	1 (25.0)	
Other	12 (0.1))	0 (0)	
**Time since fourth vaccine dose, n (%)**	**n = 15331**	**n = 4**	*-*
≤14 D	1427 (9.3)	1 (25.0)	
>14 to 60 D	5096 (33.2)	0	
>60 to 120 D	5754 (37.5)	0	
>120 to 180 D	2473 (16.1)	3 (75.0)	
>180 D	581 (3.8)	0	
**Type of fifth vaccine dose, n (%)**	**n = 394**	n = 0	*-*
Pfizer-BioNTech	60 (15.2)	0 (0)	
Moderna	334 (84.8)	0 (0)	
Median (IQR) time since last vaccination, days	105 (69–147)	127 (82–182)	*<0*.*01*
**Time since last vaccination (All schedules), n (%)**	**n = 234791**	**n = 157**	*<0*.*01*
≤14 D	7440 (3.2)	7 (4.4)	
>14 to 60 D	39623 (16.9)	14 (8.9)	
>60 to 120 D	95407 (40.6)	50 (31.9)	
>120 to 180 D	61770 (26.3)	43 (27.4)	
>180 D	30551 (13.0)	43 (27.4)	

IMV = Invasive Mechanical Ventilation, IQR = Interquartile range.

*Vaccine type missing in 1 subject.

Over half of the subjects with severe outcomes were not fully vaccinated, as compared to just 11% among those without severe outcomes. Subjects without severe outcomes were more likely to have received heterologous primary series with Sinovac/Sinopharm first dose followed by ChAdOx1 nCoV-19 second dose. No significant difference in the type of vaccine used for boosting as third, fourth and fifth dose was seen between the groups. Notably, the proportion of subjects receiving Moderna increased progressively across third (21.8%), fourth (50.1%) and fifth (84.8%) doses respectively, which is reflective of the booster dose roll-out in Thailand **(Table 1)**.

Subjects with severe outcomes had a significantly longer time lapse from date of last vaccination to positive SARS-CoV-2 test (median 127 days vs 105 days, *p<0*.*01*). Subjects without severe outcomes were more likely to have received their last vaccination between 14 and 120 days, while those with severe outcomes were more likely to have received their last vaccination >180 days prior **(Table 1)**.

The findings were consistent when subjects with death outcomes were compared with those without death outcomes **(S1 Table).**

### Waning vaccine response against severe COVID-19 outcomes and mortality

In univariate models, older age, receiving last vaccine >180 days prior to date of positive test (irrespective of vaccine series), and being unvaccinated or partially vaccinated significantly increased the risk of severe COVID-19 **(S2A, S2B and S2C Fig).**

**Fig 1:**
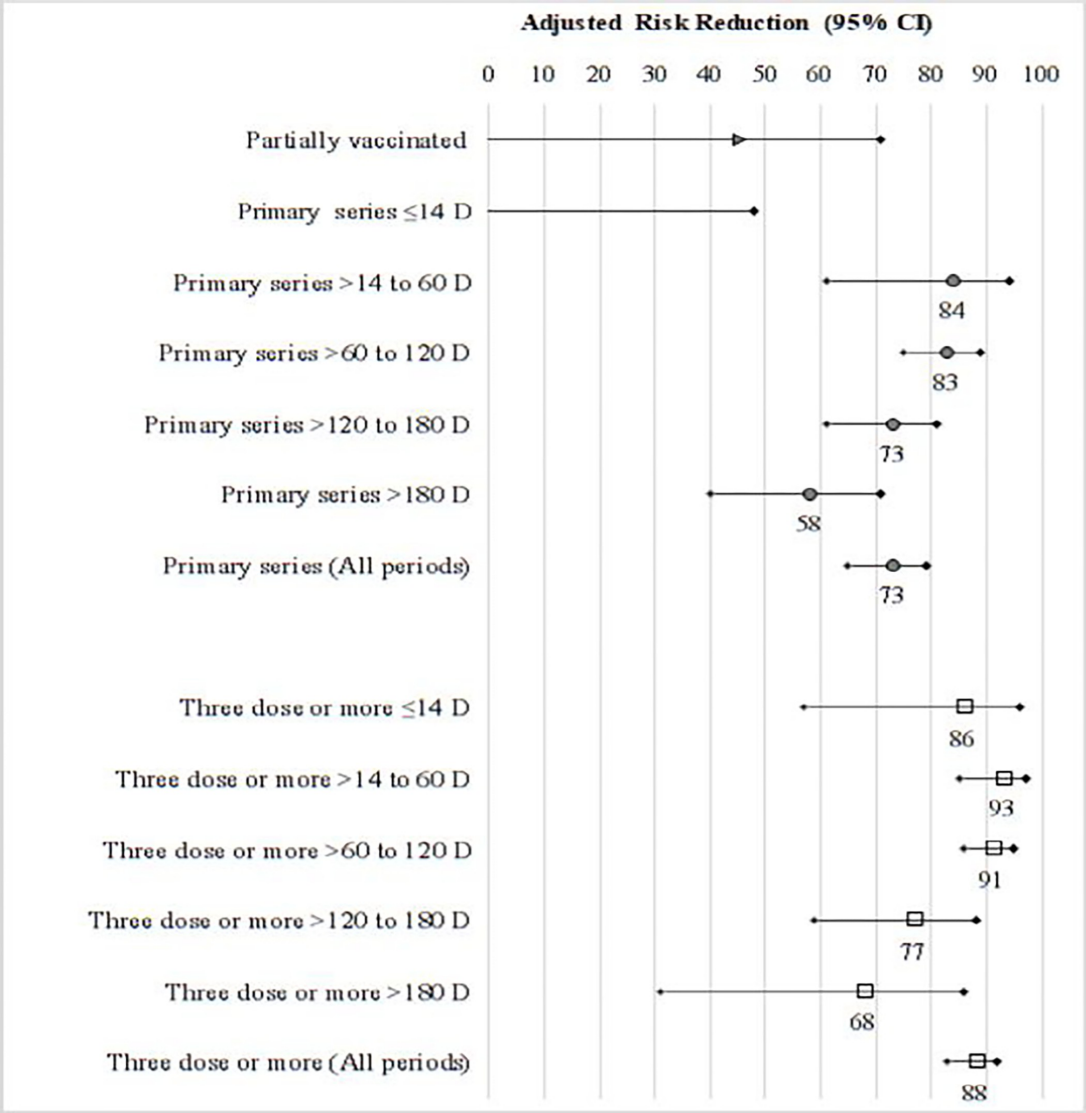
Adjusted risk reduction (95% CI) of severe COVID-19 among adults during omicron predominance, by vaccination regimens and time since last vaccine dose.

After adjusting for age, gender, and calendar time of test receiving a booster dose was associated with 88% and 90% risk reduction against severe COVID-19 and deaths respectively, as compared to unvaccinated group. The primary series was associated with a 73% and 75% reduction of risk of severe outcomes and deaths, respectively **(S2A Table, Fig 1)**.

Adjusted for age, gender and calendar week of test, Reference: Unvaccinated.

Optimal protection against both severe COVID-19 (aHR 0.07, 95% CI 0.03–0.15, *p<0*.*01*) and deaths (aHR 0.06, 95% CI 0.02–0.17, *p<0*.*01*) were observed with boosters received within >14–60 days prior to positive test **(Fig 1, S2A Table)**. Only slight waning of vaccine response was observed for boosters received within >60–120 days (aHR 0.09 for severe COVID-19 and 0.07 for deaths). As compared to booster vaccination at >14–60 days, the risk reduction declined by 16 and 25 percentage points from 120 days and 180 days onwards, respectively **(Fig 1, S2A Table)**.

Optimal protection with primary series vaccination against severe COVID-19 (aHR 0.16, 95% CI 0.06–0.39, p<0.01) and deaths (aHR 0.15, 95% CI 0.05–0.49, p<0.01) were observed with vaccines received within >14–60 days prior to positive test, and only slight waning was observed for vaccines received within >60–120 days (aHR 0.17 for severe COVID-19 and 0.18 for deaths). Waning of the response by 11 and 26 percentage points was observed from 120 days and 180 days onwards, respectively.

A sensitivity analysis which included only those who received third dose (first booster) after excluding subjects who received two or more boosters, found similar results **(S3 Fig).**

A slight difference in the long-term waning pattern was observed for deaths as compared to severe COVID-19 outcomes. While the most rapid decline in vaccine response was observed at >120 days since first positive test for both outcomes, the waning of response beyond 120 days happened at a slower pace against deaths as compared to severe COVID-19, for both primary series and booster vaccine (**S2A Table**).

### Waning vaccine response against severe COVID-19 outcomes by Age groups

**Fig 2:**
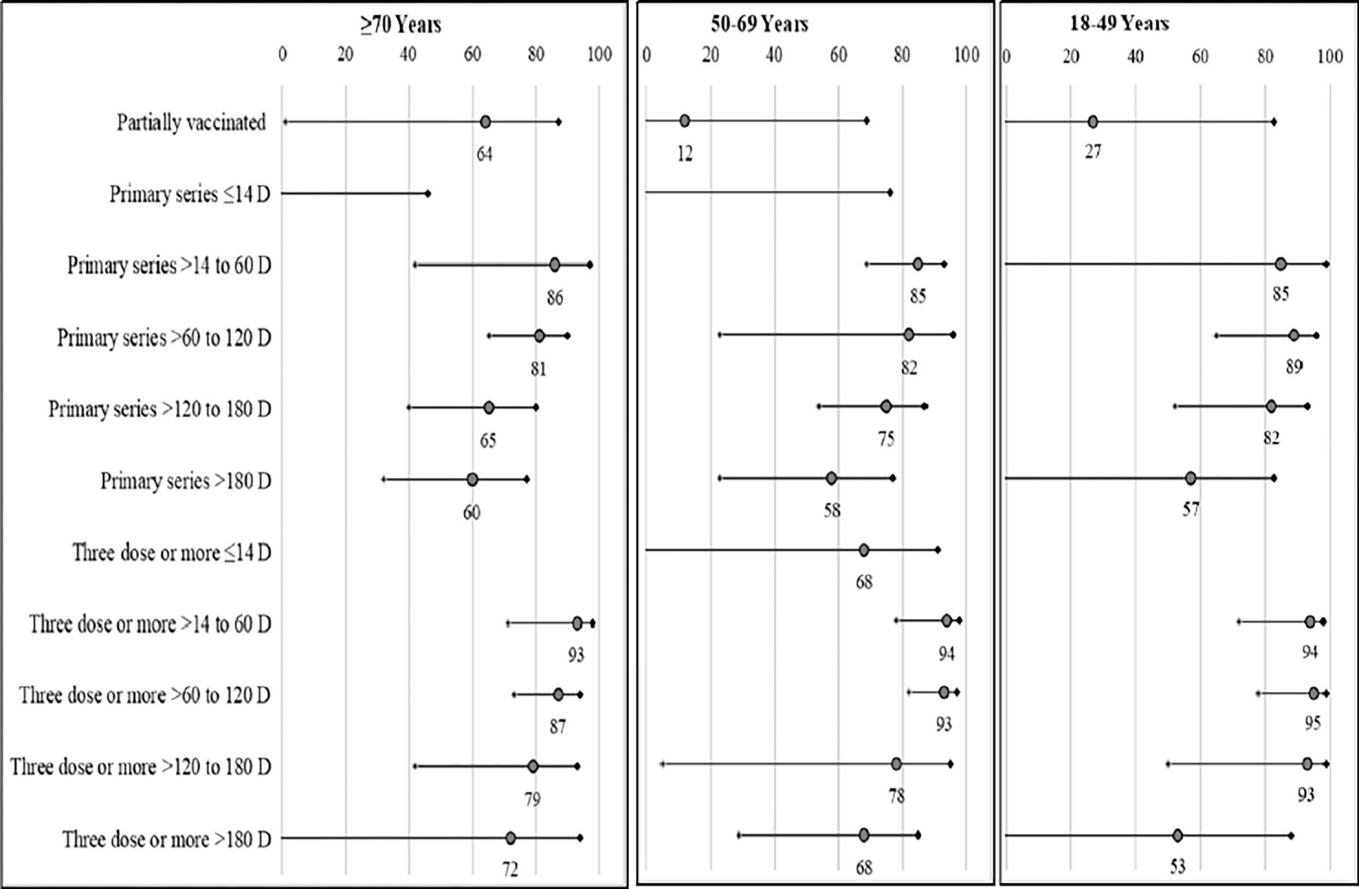
Adjusted risk reduction (95% CI) of severe COVID-19 among adults during omicron predominance, by vaccination regimens and time since last vaccine dose, stratified by age group.

We observed a difference in the waning vaccine response patterns with age for both the primary series and booster vaccine. Subjects aged 70 years or older showed waning of vaccine response as early as 60 days from the last vaccine, with a drop of ~5 percentage points for both primary series and booster vaccines. In comparison, little to no waning was seen among 50-69- and 18-49-year age groups during the same period (**Fig 2, S2B Table)**. For the ≥70- and 50–69-year age groups, the sharpest drop in vaccine response was seen >120 days from last vaccination. However, in the 18-49-year age group the protective effect lasted up to 180 days (**Fig 2, S2B Table)**.

Adjusted for age, gender and calendar week of test, Reference: Unvaccinated.

### Waning vaccine response against severe COVID-19 outcomes by type of booster vaccine

The three vaccine types used as boosters in Thailand, Pfizer-BioNTech (89%, 95% CI 72–95), ChAdOx1 nCoV-19 (81%, 95% CI 68–93%) and Moderna (82%, 95% CI 26–92), showed equivalent risk reduction against severe COVID-19 **(S3 Table, S4 Fig).** In general, the pattern of waning appears to follow that of the pooled analysis. The risk reduction with Pfizer-BioNTech was 94% within >14–60 days from last vaccine but declined by 26 percentage points to 73% by >120 days from last vaccine., In comparison, the drop in risk reduction with ChAdOx1 nCoV-19 appeared to be less rapid from 86% for vaccines received within >14–60 days to 85% at >120 days **(S4 Fig).** Readers are advised to interpret the estimates of vaccine response, particularly for Moderna, with caution due to the smaller number of events in the sub-groups.

## Discussion

While the number of COVID-19 cases and deaths globally is high, the impact of vaccinations is undisputable, when they have been implemented appropriately. Vaccination schedules have rapidly evolved to third, fourth and even fifth doses to manage new variants and concerns around waning immunity, but the availability of data to support decision makers has struggled to keep pace. The current study provides urgently needed data to understand the duration of optimal protection against severe COVID-19 outcomes with booster vaccinations and provides key data for heterologous booster dose schedules incorporating inactivated vaccines into the primary series.

We found that the protection against severe COVID-19 was highest with boosters received >14 days to 2 months prior to positive test (93%) and persisted between 2–4 months (91%) but started to wane between 5–6 months (77%) and further beyond 6 months (68%). Our results are consistent with findings from the US [16,22], Brazil [17] and Finland [18], where effectiveness of booster vaccines against COVID-19–associated severe outcomes started to decline after 4 months.

We also found that the rate of waning differed by age group. Cases who were older, particularly those aged 70 years and above, showed more rapid waning of vaccine response against severe COVID-19, while younger age groups (<50 years) retained good responses up to 6 months. Our results are in line with the findings by from Ferdinands et al. [16], and Ranzani et al.,[17], where a more rapid decline in vaccine effectiveness was seen among older adults. This evidence strengthens the need for a more targeted approach in protecting the older age groups who remain more vulnerable, either through booster vaccinations or additional preventive measures.

Our study found that the three vaccine types used for boosting in Thailand, Pfizer-BioNTech, Moderna and ChAdOx1 nCoV-19, offered similar protection against severe COVID-19 outcomes. Comparable protection from ChAdOx1 nCoV-19 and Pfizer-BioNTech against infection, hospitalization, ICU admissions and deaths, has been previously reported [12,20,23,24]. Our findings corroborate this evidence and supports the use of mRNA vaccines and viral vector vaccines as booster vaccines, providing much needed flexibility to incorporate different vaccines into schedules according to local supply and logistical considerations.

Our data strongly suggests that accelerating the booster vaccinations and increasing coverage by using any vaccines available, particularly among elderly is an important strategy to optimize protection. The authors wish to highlight a few study limitations. In the current study we were unable to examine other confounders such as chronic comorbidities which are important risk factors of severe COVID-19 outcomes and deaths. The source population were those diagnosed with COVID-19, and the testing could have been done for reasons other than signs and symptoms or clinical suspicion. We did not differentiate or control for incidental finding of COVID-19.

## Supporting information

S1 Fig Flow chart of subject selection for adult COVID-19 cases who are residents of Chiang Mai, Thailand between 1 Feb– 31 Jul 2022. (PDF)

S2 Fig a. Kaplan-Meier estimates for severe COVID-19 outcomes among adult cases during omicron predominance by age group. b. Kaplan-Meier estimates for severe COVID-19 outcomes among adult cases during omicron predominance by vaccine series. c. Kaplan-Meier estimates for severe COVID-19 outcomes among adult cases during omicron predominance by time from last vaccine.
(PDF)

S3 Fig Risk reduction of severe COVID-19 among adult cases during omicron predominance, by vaccination regimens and time since last vaccine dose (Sensitivity analysis excluding two or more boosters). (PDF)

S4 Fig Risk reduction of severe COVID-19 among adult cases during omicron predominance by booster vaccine type and time since last dose. (PDF)

S1 Table Comparison of clinical characteristics of adult COVID-19 patients with and without death outcomes during omicron predominance (1 Feb 2022–31 Jul 2022) in Chiang Mai, Thailand. (PDF)

S2 Table a. Association between vaccination regimens, time since last vaccine dose and severe COVID-19 outcomes among adult COVID-19 cases. b. Association between time since last vaccine dose and severe COVID-19 outcomes during omicron predominance among adult COVID-19 cases stratified by age group.
(PDF)

S3 Table Association between time since last vaccine dose and severe COVID-19 outcomes during omicron predominance among adult COVID-19 cases by type of booster vaccine. (PDF)
