# Supplementary material for: Waning vaccine response to severe COVID-19 outcomes during omicron predominance in Thailand
Source: PLoS One. 2023 May 11;18(5):e0284130. doi: 10.1371/journal.pone.0284130 (PMC10174527; doi:10.1371/journal.pone.0284130)

Supplementary Figure 1: Flow chart of subject selection for adult COVID-19 cases who are residents of Chiang Mai, Thailand between 1 Feb – 31 Jul 2022

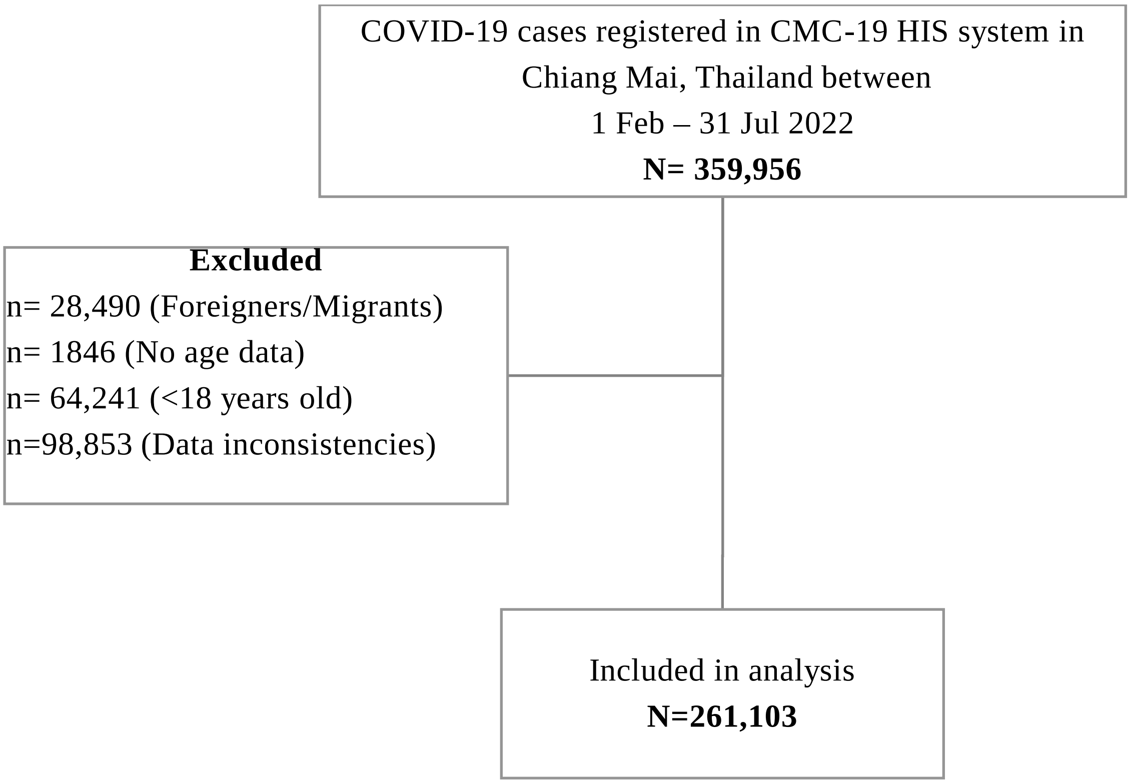

Supplement: S1 Fig — (PDF) [file pone.0284130.s001.pdf]
