# Supplementary material for: Waning vaccine response to severe COVID-19 outcomes during omicron predominance in Thailand
Source: PLoS One. 2023 May 11;18(5):e0284130. doi: 10.1371/journal.pone.0284130 (PMC10174527; doi:10.1371/journal.pone.0284130)

Supplementary Figure 2a: Kaplan-Meier estimates for severe COVID-19 outcomes among adult cases during omicron predominance by age group

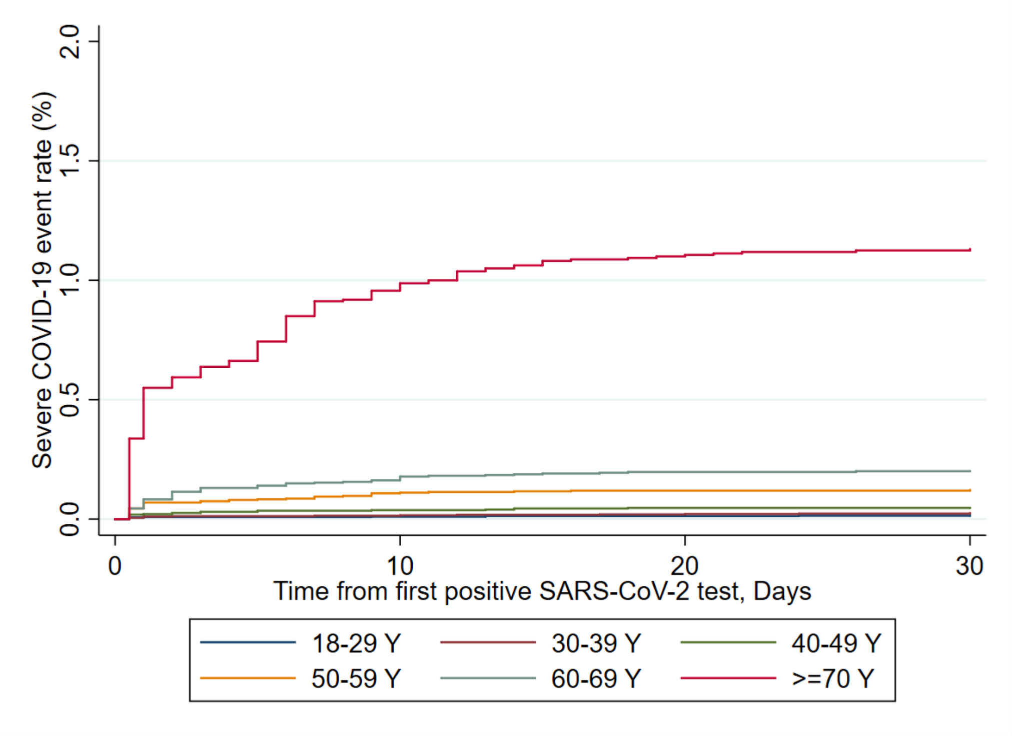

Supplementary Figure 2b: Kaplan-Meier estimates for severe COVID-19 outcomes among adult cases during omicron predominance by vaccine series

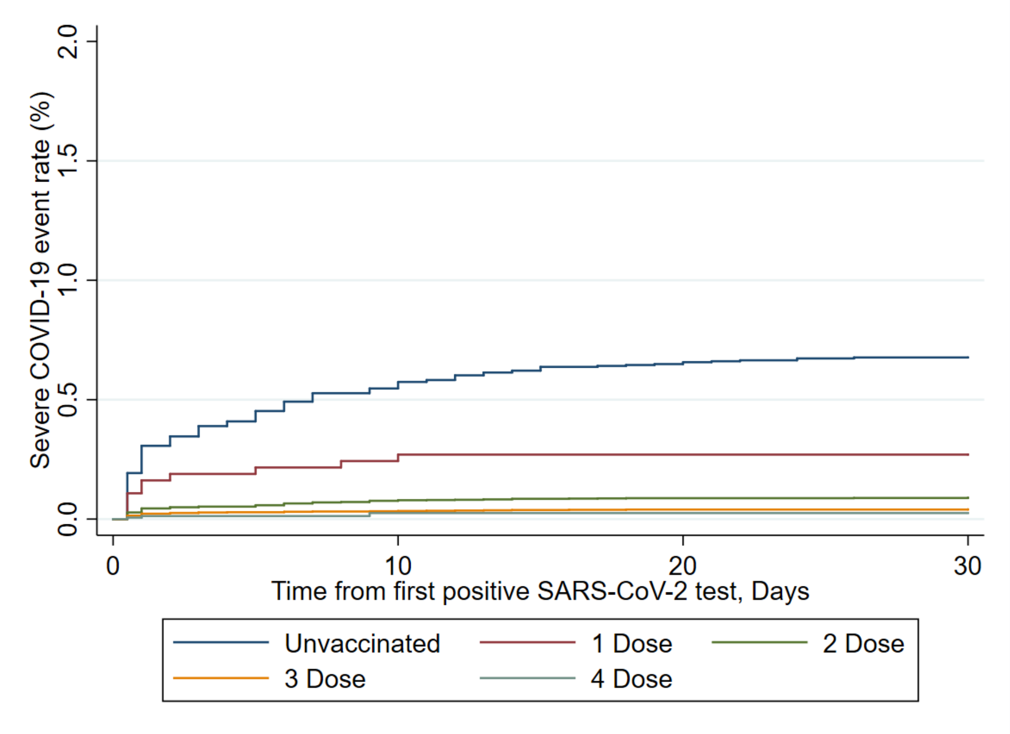

Supplementary Figure 2c: Kaplan-Meier estimates for severe COVID-19 outcomes among adult cases during omicron predominance by time from last vaccine

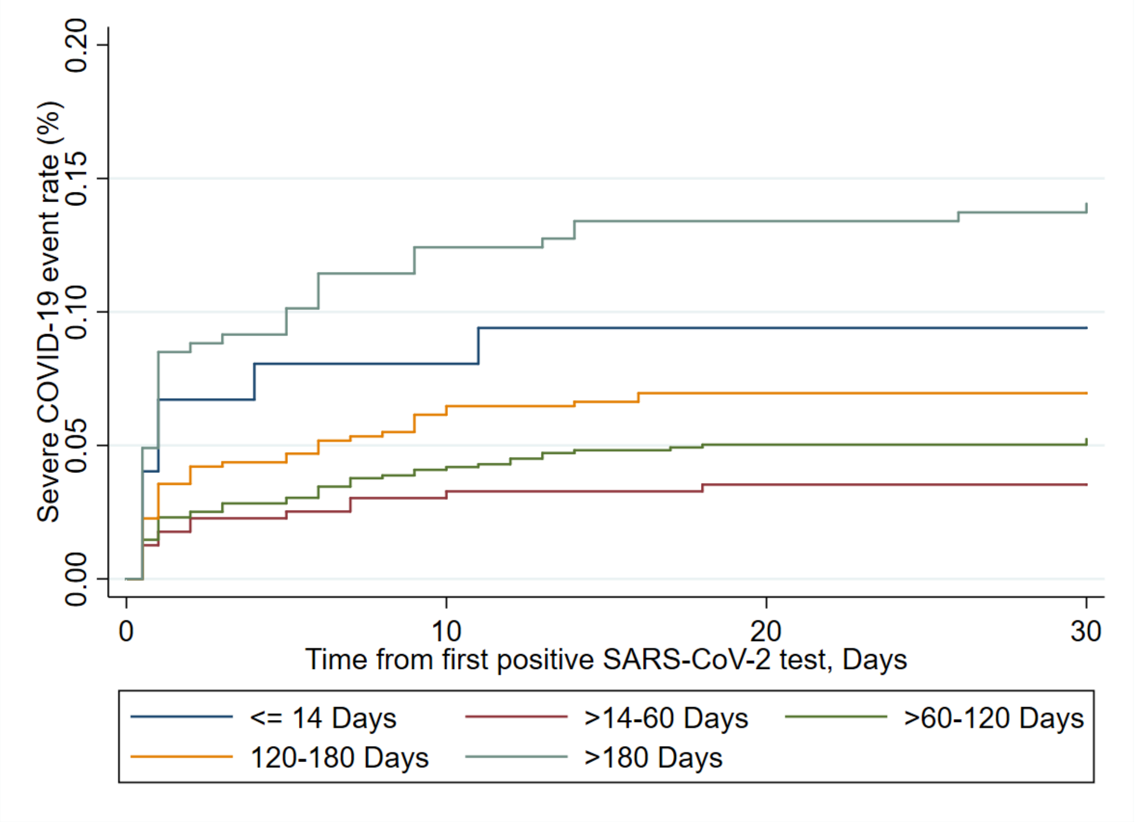

Supplement: S2 Fig — a. Kaplan-Meier estimates for severe COVID-19 outcomes among adult cases during omicron predominance by age group. b. Kaplan-Meier estimates for severe COVID-19 outcomes among adult cases during omicron predominance by vaccine series. c. Kaplan-Meier estimates for severe COVID-19 outcomes among adult cases during omicron predominance by time from last vaccine. (PDF) [file pone.0284130.s002.pdf]
