# Supplementary material for: Waning vaccine response to severe COVID-19 outcomes during omicron predominance in Thailand
Source: PLoS One. 2023 May 11;18(5):e0284130. doi: 10.1371/journal.pone.0284130 (PMC10174527; doi:10.1371/journal.pone.0284130)

Supplementary Figure 3: Risk reduction of severe COVID-19 among adult cases during omicron predominance, by vaccination regimens and time since last vaccine dose (Sensitivity analysis excluding two or more boosters)

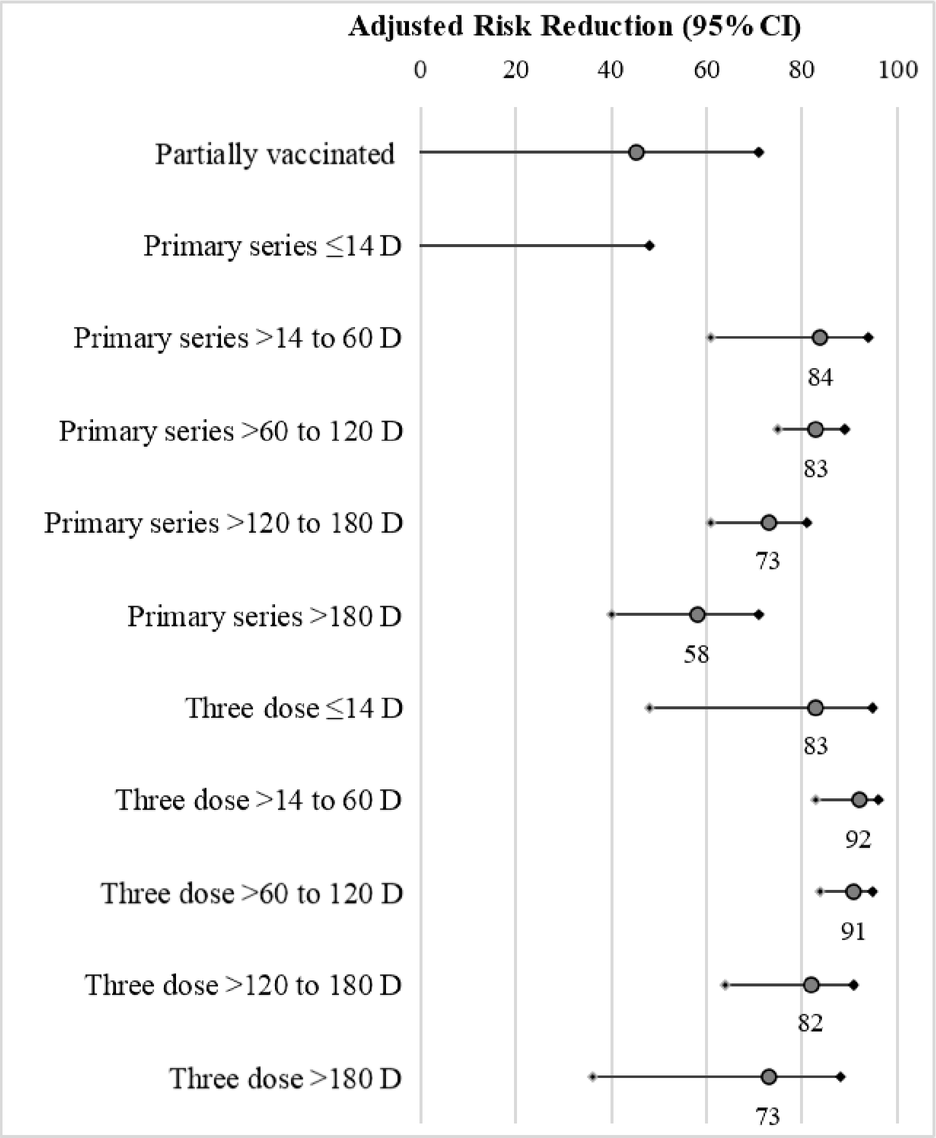

Supplement: S3 Fig — (PDF) [file pone.0284130.s003.pdf]
