# Supplementary material for: Waning vaccine response to severe COVID-19 outcomes during omicron predominance in Thailand
Source: PLoS One. 2023 May 11;18(5):e0284130. doi: 10.1371/journal.pone.0284130 (PMC10174527; doi:10.1371/journal.pone.0284130)
Supplement: S4 Fig — (PDF) [file pone.0284130.s004.pdf]

Supplementary Figure 4: Risk reduction of severe COVID-19 among adult cases during omicron predominance by booster vaccine type and time since last dose

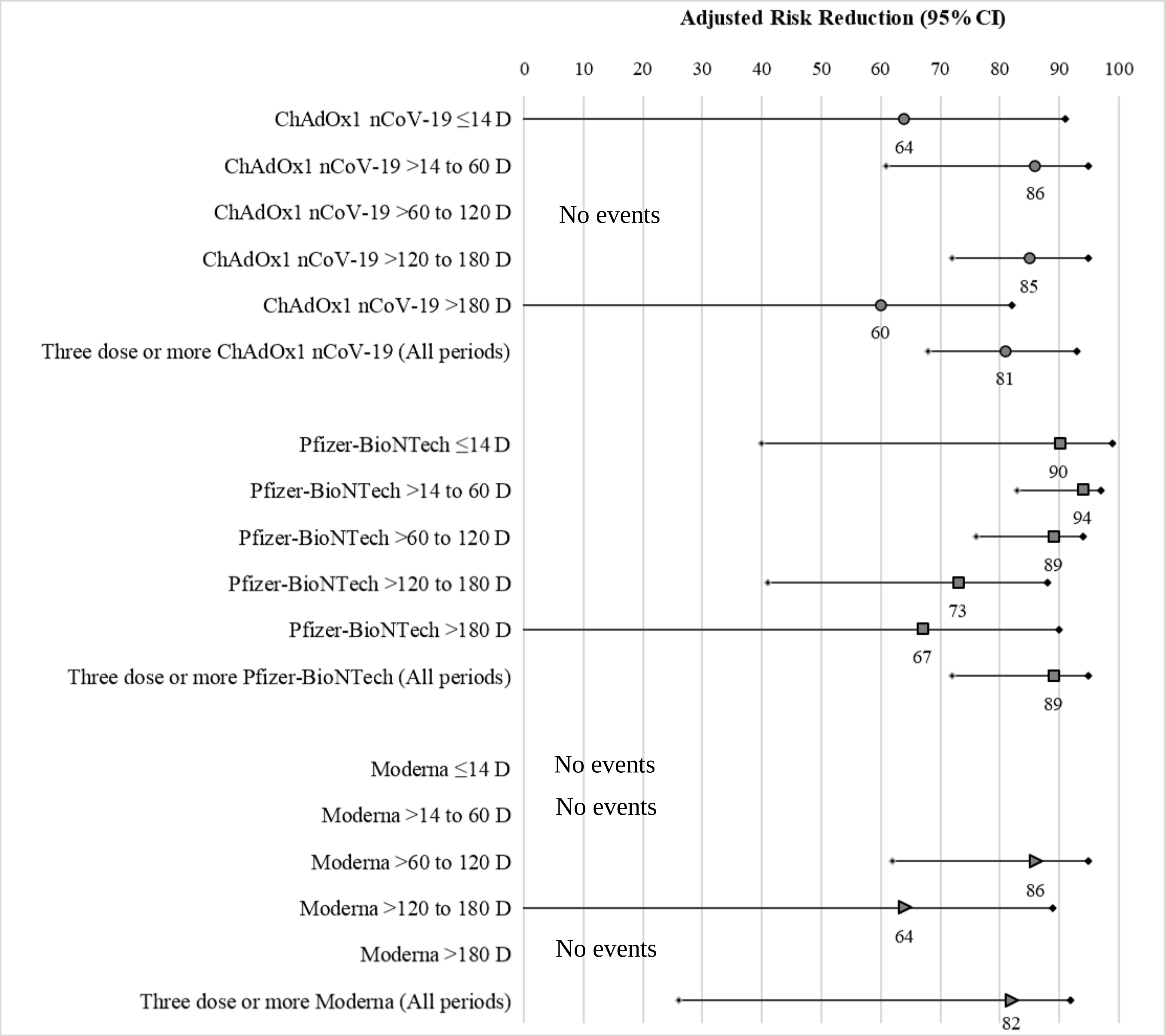

Adjusted for age, gender, calendar time and preceding vaccine series type. Reference group: Unvaccinated
