# Supplementary material for: Waning vaccine response to severe COVID-19 outcomes during omicron predominance in Thailand
Source: PLoS One. 2023 May 11;18(5):e0284130. doi: 10.1371/journal.pone.0284130 (PMC10174527; doi:10.1371/journal.pone.0284130)
Supplement: S1 Table — (PDF) [file pone.0284130.s005.pdf]

Supplementary Table 1: Comparison of clinical characteristics of adult COVID-19 patients with and without death outcomes during omicron predominance (1 Feb 2022-31 Jul 2022) in Chiang Mai, Thailand

Variable	Without death outcome	death outcome	*p-value*
**Number (%)**	260,913 (99.9)	190 (0.07)	-
**Age, years**			
Median (IQR)	39 (27-55)	72 (61-83)	*<0.01*
**Age group, n (%)**			
18-29	78819 (30.2)	9 (4.7)	
30-39	56059 (21.5)	9 (4.7)	
40-49	42681 (16.4)	8 (4.2)	*<0.01*
50-59	36062 (13.8)	19 (10.0)	
60-69	31393 (12.0)	38 (20.0)	
≥70	15899 (6.1)	107 (56.4)	
**Gender, n (%)**			
Male	115125 (44.1)	116 (61.1)	*<0.01*
Female	145788 (55.9)	74 (38.9)	
**Vaccination Status, n (%)**			
Unvaccinated	25319 (9.7)	103 (54.2)	
Partially vaccinated	3698 (1.4)	4 (2.1)	
Primary vaccine series	118721 (45.5)	61 (32.1)	*<0.01*
Vaccinated three doses	97448 (37.4)	20 (10.5)	
Vaccinated four doses or more	15727 (6.0)	2 (1.0)	
**Type of primary vaccine series, n (%)**	**n=118,721**	**n=61**	
Sinovac/Sinopharm-ChAdOx1 nCoV-19	47027 (39.6)	14 (22.9)	
Sinovac-Sinovac or Sinopharm-Sinopharm	16527 (13.9)	10 (16.4)	
ChAdOx1 nCoV-19-ChAdOx1 nCoV-19	3602 (3.0)	1 (1.6)	
Pfizer-BioNTech-Pfizer-BioNTech	13864 (11.7)	11 (18.0)	*0.11*
ChAdOx1 nCoV-19-Pfizer-BioNTech/Moderna	32126 (27.0)	20 (32.8)	
Sinovac/Sinopharm-Pfizer-BioNTech/Moderna	594 (0.5)	0 (0)	
Moderna-Moderna	4981 (4.2)	5 (8.2)	
**Type of third vaccine dose, n (%)**	**n=97448**	**n=20**	
Pfizer-BioNTech	48478 (49.7)	8 (40.0)	
ChAdOx1 nCoV-19	27620 (28.3)	7 (35.0)	*0.84*
Moderna	21266 (21.8)	5 (25.0)	
Other	84 (0.1)	0 (0)	
**Type of fourth vaccine dose*, n (%)**	**n=15332**	**n=2**	
Pfizer-BioNTech	6838 (44.6)	1 (50.0)	
ChAdOx1 nCoV-19	803 (5.2)	0 (0)	-
Moderna	7679 (50.1)	1 (50.0)	
Other	12 (0.1)	0 (0)	
**Type of fifth vaccine dose, n (%)**	**n=394**	n=0	-

Pfizer-BioNTech	60 (15.2)	0 (0)	
Moderna	334 (84.8)	0 (0)	
Median (IQR) time since last vaccination, days	105 (69-147)	120 (70-163)	*0.07*
**Time since last vaccination (All schedules), n (%)**	**n=234864**	**n=84**	
≤14 D	7442 (3.2)	5 (5.9)	*0.05*
>14 to 60 D	39630 (16.9)	7 (8.3)	
>60 to 120 D	95427 (40.6)	30 (35.7)	
>120 to 180 D	61787 (26.3)	26 (30.9)	
>180 D	30578 (13.0)	16 (19.1)	
