# Supplementary material for: Waning vaccine response to severe COVID-19 outcomes during omicron predominance in Thailand
Source: PLoS One. 2023 May 11;18(5):e0284130. doi: 10.1371/journal.pone.0284130 (PMC10174527; doi:10.1371/journal.pone.0284130)
Supplement: S2 Table — a. Association between vaccination regimens, time since last vaccine dose and severe COVID-19 outcomes among adult COVID-19 cases. b. Association between time since last vaccine dose and severe COVID-19 outcomes during omicron predominance among adult COVID-19 cases stratified by age group. (PDF) [file pone.0284130.s006.pdf]

Supplementary Table 2a: Association between vaccination regimens, time since last vaccine dose and severe COVID-19 outcomes among adult COVID-19 cases

	Severe COVID-19								Death							
	**Crude HR**	**95% CI-lower**	**95% CI-upper**	***p-value***	**Adj. HR**	**95% CI-lower**	**95% CI-upper**	***p-value***	**Crude HR**	**95% CI-lower**	**95% CI-upper**	***p-value***	**Adj. HR**	**95% CI-lower**	**95% CI-upper**	***p-value***
**Age, years**	1.09	1.08	1.09	<0.01	1.07	1.06	1.08	<0.01	1.09	1.08	1.10	<0.01	1.08	1.07	1.09	<0.01
**Gender**																
Female	Reference															
Male	1.64	1.32	2.04	<0.01	1.73	1.39	2.15	<0.01	1.98	1.48	2.65	<0.01	2.10	1.57	2.82	<0.01
**Vaccination status[1]**																
Unvaccinated	Reference															
Partially vaccinated	0.39	0.21	0.75	0.01	0.57	0.30	1.08	0.08	0.27	0.10	0.74	0.01	0.37	0.13	0.99	0.05
Primary vaccine series	0.13	0.10	0.17	<0.01	0.27	0.21	0.35	<0.01	0.13	0.09	0.18	<0.01	0.25	0.18	0.35	<0.01
Vaccinated three doses	0.06	0.04	0.08	<0.01	0.12	0.08	0.17	<0.01	0.05	0.03	0.08	<0.01	0.11	0.07	0.21	<0.01
Vaccinated four doses or more	0.04	0.01	0.10	<0.01	0.12	0.04	0.34	<0.01	0.03	0.01	0.13	<0.01	0.10	0.06	0.16	<0.01
**Vaccination status by time since last dose[1]**																
Unvaccinated	Reference								Reference							
Partially vaccinated	0.39	0.21	0.75	0.01	0.55	0.29	1.04	0.07	0.27	0.10	0.74	0.01	0.37	0.13	0.99	0.05
Primary vaccine series ≤14 D	1.01	0.32	3.16	0.98	1.64	0.52	5.17	0.39	1.16	0.28	4.72	0.83	1.72	0.42	7.01	0.44
Primary vaccine series >14 to 60 D	0.08	0.03	0.20	<0.01	0.16	0.06	0.39	<0.01	0.08	0.02	0.27	<0.01	0.15	0.05	0.49	<0.01
Primary vaccine series >60 to 120 D	0.08	0.05	0.12	<0.01	0.17	0.11	0.25	<0.01	0.10	0.06	0.16	<0.01	0.18	0.11	0.31	<0.01
Primary vaccine series >120 to 180 D	0.13	0.09	0.18	<0.01	0.27	0.19	0.39	<0.01	0.14	0.09	0.22	<0.01	0.28	0.17	0.45	<0.01
Primary vaccine series >180 D	0.24	0.17	0.35	<0.01	0.42	0.29	0.60	<0.01	0.16	0.09	0.27	<0.01	0.29	0.17	0.52	<0.01
Three dose or more ≤14 D	0.07	0.02	0.21	<0.01	0.14	0.04	0.43	<0.01	0.11	0.03	0.35	<0.01	0.22	0.07	0.71	0.01
Three dose or more >14 to 60 D	0.03	0.01	0.07	<0.01	0.07	0.03	0.15	<0.01	0.03	0.01	0.08	<0.01	0.06	0.02	0.17	<0.01
Three dose or more >60 to 120 D	0.04	0.02	0.08	<0.01	0.09	0.05	0.14	<0.01	0.04	0.01	0.08	<0.01	0.07	0.03	0.15	<0.01

Three dose or more >120 to 180 D	0.10	0.05	0.18	<0.01	0.23	0.12	0.41	<0.01	0.07	0.03	0.17	<0.01	0.20	0.08	0.47	<0.01
Three dose or more >180 D	0.14	0.06	0.29	<0.01	0.32	0.14	0.69	<0.01	0.06	0.01	0.25	<0.01	0.19	0.04	0.80	0.02

1 Adjusted for age, gender, and calendar week of test

Supplementary Table 2b: Association between time since last vaccine dose and severe COVID-19 outcomes during omicron predominance among adult COVID-19 cases stratified by age group

	≥70 Years (n=16,006)								50-69 Years (n=67,512)								18-49 Years (n=177,585)							
	**Crude HR**	**95% CI-lower**	**95% CI-upper**	***p-value***	**Adjusted HR**	**95% CI-lower**	**95% CI-upper**	***p-value***	**Crude HR**	**95% CI-lower**	**95% CI-upper**	***p-value***	**Adjusted HR**	**95% CI-lower**	**95% CI-upper**	***p-value***	**Crude HR**	**95% CI-lower**	**95% CI-upper**	***p-value***	**Adjusted HR**	**95% CI-lower**	**95% CI-upper**	***p-value***
Unvaccinated	Reference								Reference								Reference							
Partially vaccinated	0.35	0.13	0.97	0.04	0.36	0.13	0.99	0.05	0.85	0.31	2.39	0.77	0.88	0.31	2.46	0.81	0.67	0.15	2.90	0.59	0.73	0.17	3.20	0.68
Primary vaccine series ≤14 D	2.41	0.59	9.75	0.22	2.19	0.54	8.90	0.27	1.83	0.25	13.30	0.54	1.76	0.24	12.90	0.57	No events							
Primary vaccine series >14 to 60 D	0.13	0.03	0.53	<0.01	0.14	0.03	0.58	0.01	0.15	0.07	0.31	<0.01	0.15	0.07	0.31	<0.01	0.13	0.01	0.98	0.05	0.15	0.01	1.17	0.07
Primary vaccine series >60 to 120 D	0.17	0.09	0.30	<0.01	0.19	0.10	0.35	<0.01	0.18	0.04	0.77	0.02	0.18	0.04	0.77	0.02	0.10	0.03	0.30	<0.01	0.11	0.04	0.35	<0.01
Primary vaccine series >120 to 180 D	0.28	0.17	0.48	<0.01	0.35	0.20	0.60	<0.01	0.24	0.13	0.44	<0.01	0.25	0.13	0.46	<0.01	0.17	0.07	0.44	<0.01	0.18	0.07	0.48	<0.01
Primary vaccine series >180 D	0.31	0.18	0.51	<0.01	0.40	0.23	0.68	<0.01	0.39	0.22	0.71	<0.01	0.42	0.23	0.77	0.01	0.41	0.17	0.98	0.04	0.43	0.17	1.09	0.08
Three dose or more ≤14 D	No events								0.32	0.09	1.03	0.05	0.32	0.09	1.05	0.06	No events							
Three dose or more >14 to 60 D	0.05	0.14	0.22	<0.01	0.07	0.02	0.29	<0.01	0.06	0.02	0.21	<0.01	0.06	0.02	0.22	<0.01	0.06	0.01	0.30	<0.01	0.06	0.02	0.28	<0.01
Three dose or more >60 to 120 D	0.09	0.04	0.21	<0.01	0.13	0.06	0.27	<0.01	0.07	0.03	0.17	<0.01	0.07	0.03	0.18	<0.01	0.05	0.01	0.23	<0.01	0.05	0.01	0.22	<0.01
Three dose or more >120 to 180 D	0.14	0.05	0.39	<0.01	0.21	0.07	0.58	<0.01	0.18	0.04	0.74	0.02	0.22	0.05	0.95	0.04	0.07	0.01	0.49	0.01	0.07	0.01	0.50	0.01
Three dose or more >180 D	0.19	0.05	0.76	0.02	0.28	0.06	1.17	0.08	0.20	0.13	0.59	<0.01	0.32	0.15	0.71	0.01	0.48	0.14	1.66	0.25	0.47	0.12	1.76	0.26

Adjusted for age, gender, and calendar week of test
