# Supplementary material for: Waning vaccine response to severe COVID-19 outcomes during omicron predominance in Thailand
Source: PLoS One. 2023 May 11;18(5):e0284130. doi: 10.1371/journal.pone.0284130 (PMC10174527; doi:10.1371/journal.pone.0284130)
Supplement: S3 Table — (PDF) [file pone.0284130.s007.pdf]

Supplementary Table 3: Association between time since last vaccine dose and severe COVID-19 outcomes during omicron predominance among adult COVID-19 cases by type of booster vaccine

	**n**	**Crude HR**	**95% CI-lower**	**95% CI-upper**	***p-value***	**Adjusted HR**	**95% CI-lower**	**95% CI-upper**	***p-value***
ChAdOx1 nCoV-19 ≤14 D	1327	0.22	0.05	0.89	0.03	0.36	0.09	1.45	0.15
ChAdOx1 nCoV-19 >14 to 60 D	7311	0.08	0.03	0.21	<0.01	0.14	0.05	0.39	<0.01
ChAdOx1 nCoV-19 >60 to 120 D	11363	No events							
ChAdOx1 nCoV-19 >120 to 180 D	5693	0.07	0.02	0.24	<0.01	0.15	0.05	0.28	<0.01
ChAdOx1 nCoV-19 >180 D	2736	0.21	0.08	0.58	<0.01	0.40	0.18	1.38	0.18
One or more booster ChAdOx1 nCoV-19	28430	0.10	0.05	0.32	<0.01	0.19	0.07	0.32	<0.01
Pfizer-BioNTech ≤14 D	2948	0.05	0.01	0.35	0.03	0.10	0.01	0.60	0.01
Pfizer-BioNTech >14 to 60 D	16350	0.03	0.01	0.08	<0.01	0.06	0.03	0.17	<0.01
Pfizer-BioNTech >60 to 120 D	24120	0.06	0.03	0.12	<0.01	0.11	0.06	0.24	<0.01
Pfizer-BioNTech >120 to 180 D	8503	0.12	0.06	0.26	<0.01	0.27	0.12	0.59	<0.01
Pfizer-BioNTech >180 D	3465	0.13	0.04	0.39	<0.01	0.33	0.10	1.04	0.06
One or more booster Pfizer-BioNTech	55386	0.08	0.03	0.28	<0.01	0.11	0.05	0.28	<0.01
Moderna ≤14 D	2408	No events							
Moderna >14 to 60 D	9479	No events							
Moderna >60 to 120 D	11651	0.05	0.02	0.13	<0.01	0.14	0.05	0.38	<0.01
Moderna >120 to 180 D	4473	0.09	0.03	0.31	<0.01	0.36	0.11	1.15	0.08
Moderna >180 D	1274	No events							
One or more booster Moderna	29285	0.07	0.03	0.23	<0.01	0.18	0.08	0.74	<0.01

Adjusted for age, gender, calendar time and preceding vaccine series type. Reference group: Unvaccinated
